# A direct comparison of liquid chromatography-mass spectrometry with clinical routine testing immunoassay methods for the detection and quantification of thyroid hormones in blood serum

**DOI:** 10.1007/s00216-019-01724-2

**Published:** 2019-05-11

**Authors:** Samantha L. Bowerbank, Michelle G. Carlin, John R. Dean

**Affiliations:** Department of Applied Sciences, Northumbria University, Ellison Building, Newcastle upon Tyne, NE1 8ST UK

**Keywords:** Liquid chromatography-mass spectrometry, Immunoassays, Thyroid hormones, Serum, Quantitative analysis

## Abstract

A new and improved method was developed for the determination and quantification of four “free” thyroid hormones (i.e. 3,5-diiodothyronine (T2), 3,3′,5-triiodothyronine (T3), 3,3′,5′-triiodothyrone (rT3) and 3,5,3′,5′-tetraiodothyronine (T4)) in human serum by low- and high-resolution liquid chromatography-mass spectrometry (LC-MS). Several sample preparation strategies were investigated to obtain matrix-independent results. These strategies included solid phase extraction and matrix dilution. The developed analytical methods were then directly compared, in a blind study using patient-derived human blood serum samples, to the current clinical routine testing methods, i.e. electrochemiluminescence immunoassay and enzyme-linked immunosorbent assay. Chromatographic separation was achieved on a pentafluorophenyl (F5) column with an isocratic method of 30% aqueous phase, 70% organic phase where mobile phase A is 0.1% formic acid in water (pH 4) and mobile phase B is 0.1% formic acid in methanol (pH 4) (*v*/*v*). The high-resolution LC-MS was able to give a significant improvement in sensitivity with limits of quantification of 0.002 to 0.008 pmol/L for all four “free” thyroid hormones, as well as reduced sample preparation, making this the preferred method. However, the increase in capital cost may be beyond the capabilities of some laboratories. The LC-MS methods allow for the analysis of “free” thyroid hormones to be carried out in a significantly reduced analysis time. Clinical sample analysis showed that there was no statistical difference between the results obtained by ECLIA/ELISA and both LC-MS methods.

**Electronic supplementary material:**

The online version of this article (10.1007/s00216-019-01724-2) contains supplementary material, which is available to authorized users.

**Figure:**
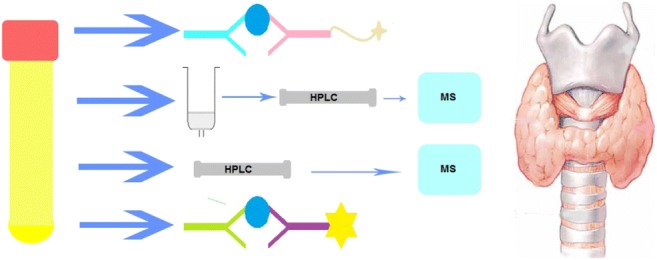
Graphical abstract

## Introduction

**Fig. 1:**
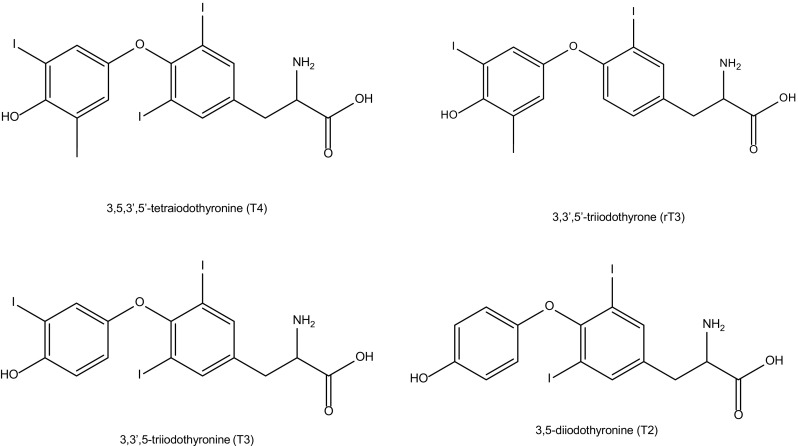
Structures of thyroid hormones T4, T3, rT3 and a T2 isomer

Thyroid compounds are a group of hormones responsible for the regulation of a variety of biological functions, including basal metabolic rate, lipid, glucose and carbohydrate metabolism [1]. This group of compounds contains tyrosine-based compounds including the physiologically active form triiodothyronine (T3). The majority of triiodothyronine is formed enzymatically by the deiodination of thyroxine (T4) [2, 3]. T4 can also be deiodinated to form an inactive form of T3 called reverse-triiodothyronine (rT3). Then, the physiologically active T3 is further metabolised to form one of the isomers of T2, i.e. 3,5-diiodothyroxine, prior to elimination from the body (Fig. 1). Normal thyroid hormone reference intervals have a large degree of variability based on a number of factors including age, sex and ethnic origin of the population; the range is calculated based on a 2.5th to 97.5th percentile of a normal population with a seemingly healthy thyroid function [4–6]. Within the Newcastle upon Tyne NHS Trust, the reference ranges are 3.5–6.5 pmol/L and 9.5–21.5 pmol/L for T3 and T4, respectively [7, 8].

There are two main medical conditions associated with thyroid hormone levels: hyperthyroidism and hypothyroidism. Hyperthyroidism is caused by an elevated level of triiodothyronine and is clinically indicated by a low thyroid-stimulating hormone level and an elevated T4 and/or T3 concentration. Symptoms of which includes excessive weight loss, heat intolerance, tremor, rapid heart rate and palpitations and if left untreated can cause heart failure, osteoporosis, eye problems and miscarriage [9]. Hypothyroidism is caused by a depleted level of triiodothyronine and is clinically indicated by a high-normal thyroid-stimulating hormone level and a depleted T4 level. Symptoms of which include inability to lose weight and lack of energy [1, 10–13].

**Fig. 2:**
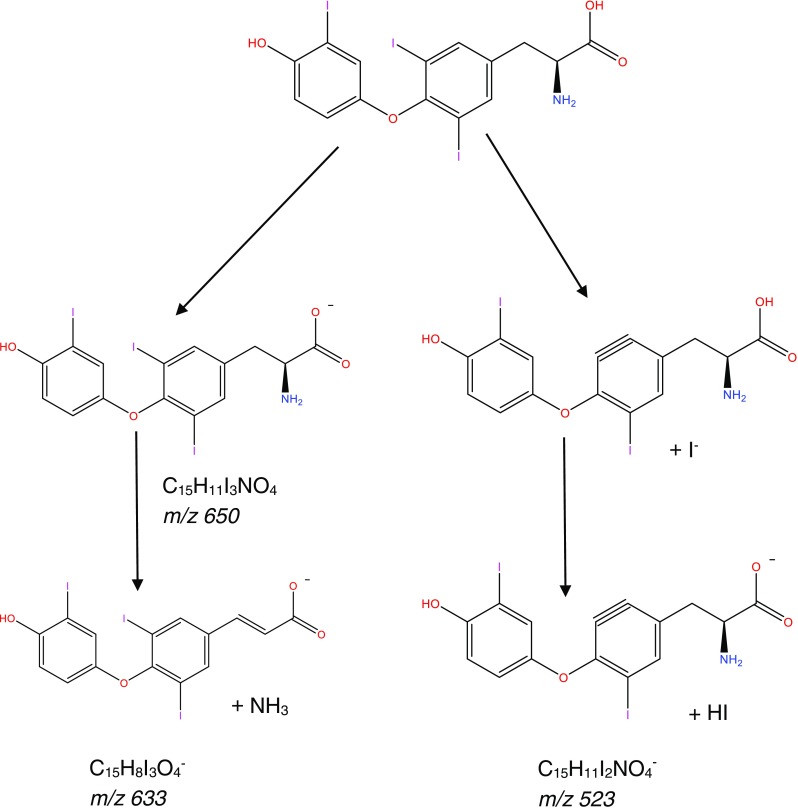
Mass spectrum fragmentation for T3

Currently, patient samples are analysed utilising electrochemiluminescent immunoassay (ECLIA); however, each hormone must be detected separately with an analysis time of 18 min per hormone [14]. ECLIA uses electrochemiluminescent labelled molecules, such as tris(2,2′-bipyridyl)ruthenium(II) complex, which is repeatedly excited with tripropilamine resulting in an amplification of the light signal which is detected [14]. The sensitivity of this technique also has an impact on the diagnosis and management of hypothyroidism samples from hypothyroid patients as hormone levels are often below the limits of quantification. Both of which can cause significant problems when monitoring thyroid hormone levels particularly in pregnant women [15–17]. The method sheets supplied by the manufacturer state a permittable error at the LOQ level of 30% has the potential to cause inaccuracies in biological sample with lower thyroid level leading to misleading results [18, 19]. Alternatively, research samples are often analysed by enzyme-linked immunosorbent assay (ELISA) due to unavailability of specialised instrumentation. However, ELISA shows limited sensitivity for both thyroxine and triiodothyronine [20, 21] and these limitations have led to a number of studies into alternative analytical techniques. More recent research have focused on utilising liquid chromatography coupled to a mass spectrometer (LC-MS) in order to determine a method for the simultaneous analysis of thyroid-stimulating hormone (TSH), T4 and T3 [22–24]. One potential problem with these methods is the separation of 3,5,3′-triiodothyronine (T3) and 3,3′,5′-triiodothyronine (rT3), due to the high level of structural similarity between the compounds. However, as rT3 is not physiologically active, it is necessary to be able to separate the two compounds in order to obtain an accurate result [2]. A study found that rT3 and T3 can be separated when the MS detector is operated in negative mode [25]. This is due to the production of a difference in the mass spectrum produced for the two compounds under negative ionisation MS/MS conditions [25]. Allowing for monitoring of *m*/*z* 649.6, 632.5 and 522.9 for T3 and *m*/*z* 649.6, 632.5 and 478.5 for rT3 (see Fig. 2 for an example MS fragmentation for T3) [26].

In addition, LC-MS instrumentation is becoming more common place in clinical biochemistry laboratories: a technique which would lend itself well to the simultaneous analysis of thyroid hormones while also reducing the analysis time. Due to the structural similarities between the four compounds, particularly T3 and rT3, more sophisticated column chemistries are required to give adequate resolution with a suitable run time. It has been well reported that phenyl-based columns offer an additional retention mechanism by introducing the possibility of *π*–*π* interactions. Pentafluorophenyl column have been found to give increased resolution capacity with short run times [27–30]. Acetonitrile has previously been found to impede *π*–*π* interactions between the analytes and the phenyl groups found on the stationary phase. Methanol, however, has been shown to improve this selectivity [31] and is therefore a most suitable organic modifier than acetonitrile when using phenyl-based columns.

There are a number of papers documenting the development and comparison of LC-MS methods for the analysis of thyroid hormones, utilising positive ionisation. These include the thyroid hormones T4 and/or T3 [32–34] as well as rT3 and T2 [35]. Typical sample preparation extraction recoveries, for the thyroid hormones, ranged between 62.7 and 114% with a coefficient of variation of 1.8–31% and precision between 3.8 and 12% [35]. Correlation between LC-MS and immunoassay methods, resulting in *r*^2^ values between 0.30 and 0.90 [32–35]. To be able to accurately measure T3 levels, in blood serum, it is necessary to ensure that rT3 does not skew the data. Therefore, rT3 is included within the method development to ensure chromatographic separation. Advantageously, both rT3 and T3 yield a different fragmentation pattern when the mass spectrometer is operated in negative mode allowing for the monitoring of different product ions [25]. In a similar study, a comparison between LC-MS/MS and immunoassay-based methods has been done on human serum, urine and post-mortem blood for cannabinoids, opiates, amphetamines, cocaine, benzodiazepines and methadone [36]. The developed LC-MS/MS method [36] was considered to be an effective screening method for forensic purposes in different matrices, based on a simple protein precipitation strategy.

This paper investigates the simultaneous analysis of “free” thyroid hormones using LC-MS in blood serum; “free” thyroid hormones are defined as those that are not protein-bound in the blood. Our analytical capabilities allow analysis of “free” thyroid hormones by low resolution, using an ion trap mass spectrometer, and high resolution using an orbitrap mass spectrometer. The results are compared, in a blind study, with samples run using clinical routine testing methods based on immunoassay (ECLIA and ELISA). The benefits of the LC-MS approaches are compared.

## Experimental

### Chemicals and reagents

Standards of T4, T3, rT3 and T2 were purchased from Sigma-Aldrich (Poole, Dorset) with a purity of ≥ 98%. Organic LC-MS grade solvents, methanol, acetic acid and formic acid were purchased from Sigma-Aldrich (Poole, Dorset). Hypersep™ C18 100 mg solid phase extraction (SPE) cartridges were purchased from Thermo Fisher Scientific (Hemel Hempstead, UK). Nylon 0.2 μm syringe filters were purchased from Thames Restek (High Wycombe, UK). Horse serum was purchased from TCS Biosciences (Buckingham, UK). Previously tested human blood serum samples were obtained from the Royal Victoria Infirmary, North Tyneside NHS trust. All biological samples were stored at − 80 °C prior to analysis.

### Instrumentation

For low-resolution LC-MS analysis, chromatographic separation was achieved using a Thermo Surveyor LC (Thermo Scientific, Hemel Hempsted, UK) consisting of a quaternary MS pump, vacuum degasser, a thermostated autosampler (set to 5 °C) and a thermostated column oven (set to 25 °C). Mass spectrometry was performed using a LTQ XL ion trap mass spectrometer (Thermo Scientific, Hemel Hempsted, UK) equipped with a heated electrospray ionisation (HESI) source maintained at 200 °C. The solvent evaporation was aided with auxiliary gas, sheath gas and sweep gas set to an arbitrary flow rate of 15, 60 and 1, respectively. The mass spectrometer was operated in selected reaction monitoring (SRM) MS/MS in negative mode; collision energies and monitoring ions are shown in Table 1. The monitored transitions were 776 → 604, 650 → 633, 650 → 479 and 524 →507 for T4, T3, rT3 and T2, respectively. In SRM MS/MS mode, the precursor ion is isolated and subjected to a specified amount of collision energy to induce fragmentation. The MS method is then set to monitor the precursor and a minimum of two stable product ions. Chromatographic separation was achieved on a reversed phase pentafluorophenyl column (Supelco 2.1 μm F5, 100 × 2.1 mm) purchased from Sigma-Aldrich. Sample aliquots of 10 μL were introduced onto the column at a flow rate of 200 μL/min. The analytes were separated using water + 0.2% formic acid (A) and methanol + 0.2% formic acid (B) as the mobile phase.

**Table 1:** Analytical figures of merit for low- and high-resolution LC-MS and Immunoassay methods

Compound	Technique	Calibration range (pmol/L)	No. of data points	Linearity *y* = *mx* + *c*	*R*^2^ value	Precision^a,b^ (%CV)		LOD (pmol/L)		LOQ (pmol/L)		*m*/*z*	Monitored transition mass (*m*/*z*)	Collision energy (eV)
						Average standard^c^	Sample	Standard	Sample	Standard	Sample			
T4	LR-LC-MS	0–257	11	33.834*x* + 1.2857	0.9996	3.3 (3.8; 3.4; 2.6)	3.3	1.76	1.8	5.9	5.5	776	776 → 759, 604^d^	28
	HR-LC-MS	0–129	11	128,022*x* + 376.6945	0.9965	3.9 (4.2; 3.8; 3.6)	3.8	0.0014	0.001	0.0045	0.0064			25
	ECLIA	0–100	NR	1.02*x* − 0.983	0.9960	NR	3.0	0.5	0.5	3	3	NA	NA	NA
	ELISA	0–50	8	− 0.215In(*x*) + 0.9397	0.9756	NR	5.0	NR	NR	2.9	2.9	NA	NA	NA
T3	LR-LC-MS	0–307	11	59.937*x* − 13.196	0.9998	2.1 (2.8; 1.4; 2.1)	2.2	0.052	0.046	0.172	0.154	650	650 → 633^d^, 523	27
	HR-LC-MS	0–154	11	264,700*x* − 65.667	0.9922	1.8 (2.5; 1.7; 1.2)	1.8	0.0017	0.0015	0.0058	0.0061			23
	ECLIA	0–50	NR	1.0 *x* − 0.107	0.9980	NR	3.1	NR	0.6	NR	1.5	NA	NA	NA
	ELISA	0–5	8	− 0.165In(*x*) + 1.6212	0.9956	NR	9.9	NR	NR	NR	3.7	NA	NA	NA
rT3	LR-LC-MS	0–307	11	53.737*x* + 46.81	0.9918	2.2 (3.0; 2.1; 1.4)	2.3	0.16	0.2	0.582	0.6	650	650 → 633, 479^d^	27
	HR-LC-MS	0–154	11	127,180*x* − 104.6175	0.9944	3.2 (3.9; 2.7; 3.0)	3.0	0.0018	0.002	0.006	0.008			23
T2	LR-LC-MS	0–367	11	38.461*x* + 42.429	0.9912	3.2 (4.0; 2.2; 3.3)	3.2	0.05	0.04	0.168	0.17	524	524 → 507^d^, 397	29
	HR-LC-MS	0–183	11	128,270*x* − 202.8385	0.9901	1.8 (2.4; 1.6; 1.4)	1.9	0.0004	0.0006	0.0015	0.002			25

*NR* not reported, *ECLIA* electrochemiluminescent immunoassay, *ELISA* enzyme-linked immunosorbent assay, *LR-LC-MS* low-resolution liquid chromatography-mass spectrometry, *HR-LC-MS* high-resolution liquid chromatography-mass spectrometry

^a^Intermediate precision over the duration of the project was determined (T2 LR-LC-MS 2.8%CV and T2 HR-LC-MS 2.1%CV; rT3 LR-LC-MS 2.4%CV and rT3 HR-LC-MS 3.0%CV; T3 LR-LC-MS 2.0%CV and T3 HR-LC-MS 1.9%CV; T4 LR-LC-MS 3.5%CV and T4 HR-LC-MS 3.6%CV)

^b^Instrument precision: LR-LC-MS 0.3%CV and HR-LC-MS 0.2%CV

^c^Values in brackets are the low, mid-point and high calibration data precision

^d^Ion used for quantitation

For high-resolution LC-MS analysis chromatographic separation was achieved using a Thermo Scientific ultimate 3000 LC (Thermo Scientific, Hemel Hempsted, UK) consisting of a quaternary MS pump, a thermostated autosampler (set to 5 °C) and a thermostated column oven (set to 25 °C). Mass spectrometry was performed using a Q-Exactive hybrid orbitrap mass spectrometer (Thermo Scientific, Hemel Hempsted, UK) equipped with a heated electrospray ionisation (HESI) source maintained at 200 °C. The solvent evaporation was aided with auxiliary gas, sheath gas and sweep gas set to an arbitrary flow rate of 15, 60 and 1, respectively. The mass spectrometer was operated in SRM MS/MS in negative mode; collision energies and monitoring ions are shown in Table 1.

Chromatographic separation was achieved on a reversed phase pentafluorophenyl column (Supelco 2.1 μm F5, 100 × 2.1 mm) purchased from Sigma-Aldrich. Sample aliquots of 10 μL were introduced onto the column at a flow rate of 200 μL/min. The analytes were separated using water + 0.2% formic acid (A) and methanol + 0.2% formic acid (B) as the mobile phase.

Electrochemiluminescence immunoassay for both T3 and T4 was performed using a Cobas e602 immunoassay analyser (Roche Diagnostics Ltd., Burgess Hill, UK). All reagents were purchased as an analysis kit from Roche Diagnostics Ltd. (Burgess Hill, UK). A 15-μL aliquot of sample is analysed via automated assay consisting of two incubation steps followed by chemiluminescent emission measurement [18, 19].

Enzyme-linked immunosorbent assay for T3 and T4 was performed using commercially available competitive ELISA kits containing all reagent from Thermo Scientific (Hemel Hempsted, UK) and measured using a BioTek microtitre plate reader set to 450 nm (Swindon, UK). To a microcentrifuge tube, 5 μL of sample and 5 μL of dissociation reagent were added and vortexed gently prior to 5 min incubation at room temperature and then 90 μL of 1× assay buffer was added. Calibration standards were prepared as per the kit instructions. To the appropriate wells, 10 μL of sample or standard was added followed by 25 μL of conjugate and 25 μL of antibody. The plate was then mixed and covered prior to 1 h incubation at room temperature with shaking. Following incubation, the solution was aspirated from each well and the wells were washed four times with 300 μL of wash buffer, then 100 μL of TMB substrate was added and incubated for further 30 min. To each well, 50 μL of stop solution was added and the plate was read at 450 nm within 10 min [20, 21].

### Preparation of stock solutions and samples

Stock solutions of each hormone were prepared at a concentration of 0.5 mg/mL in methanol and aliquoted into 100 μL aliquots and stored at − 20 °C. Calibration standards were prepared daily for each analysis from the stock solution by diluting in mobile phase (unless otherwise stated). Calibration standards were prepared over a concentration range of 0–257, 0–307 and 0–367 pmol/L for low-resolution LC-MS and 0–129, 0–154 and 0–183 pmol/L for high-resolution LC-MS for T4, T3/rT3 and T2 respectively. A full set of calibration standards were ran at both the start and end of each chromatographic run cycle. In addition, a quality control standard was prepared at 50 pmol/L, by diluting stock standard solutions in mobile phase and analysed at points throughout the run to ensure there was no deviation throughout the run. The concentration of the quality control was selected as it is a mid-reference range for T4 [19].

Matrix-matched calibration standards for LOQ and LOD determination were prepared in triplicate from the stock solution by diluting in horse serum and extracted by SPE. Calibration standards were prepared over a concentration range of 0.5–200 pmol/L for low-resolution LC-MS and 0.001–100 pmol/L for high-resolution LC-MS.

For low-resolution LC-MS serum samples were thawed and vortexed prior to extraction. The final developed method was applied to samples, prepared in triplicate, by transferring 500 μL to microcentrifuge tubes along with 500 μL of 0.1% formic acid in water. Samples were vortexed and centrifuged at 5300*g* for 5 min. The supernatants were loaded onto a HyperSep™ C18 solid phase extraction (SPE) cartridge, which were preconditioned sequentially with 2 mL of 0.1% formic acid in methanol and 2 mL 0.1% formic acid in water. The cartridge was washed with 1 mL of 30% methanol in water and then the target compounds were eluted with 1 mL of 80% methanol in water. The eluent was dried down under a stream of nitrogen and reconstituted in 50 μL of mobile phase.

For high-resolution LC-MS, serum samples were thawed and vortexed prior to dilution. Samples were prepared by transferring 10 μL of sample to an autosampler vial and diluted with 990 μL of water and vortex mixed.

### Method validation

The developed method was validated in accordance with the EMA guidelines [37]. Horse serum was used as a blank matrix due to having much lower levels of thyroid hormones than human samples. With normal ranges of 0.00024–0.00128 pmol/L and 0.0165–0.0244 pmol/L found in adult horses compared with that of 3.1–6.8 pmol/L and 12–22 pmol/L for adult humans for T3 and T4, respectively [18, 19, 38]. Furthermore, the levels in horse serum are below the limit of detection for both the low- and high-resolution instruments. Selectivity was determined by spiking thyroid hormones into horse serum at a concentration of 50 pmol/L followed by replicate analyses of blank horse serum. This solution was extracted following both the low- and high-resolution samples procedures and analysed in full scan mode to identify any potentially interfering matrix components. To access for any potential ion suppression or enhancement, thyroid hormones at low, mid and high concentrations were spiked into extracted horse serum and compared against standard peak area. Linearity was determined by plotting peak area against concentration. An *r*^2^ value of > 0.99 was deemed to show linearity. Limits of detection (LOD) and quantification (LOQ) was determined using replicate preparation of the calibration curve. The slope of the curve and the standard deviation of the intercept were used to calculate the LOD and LOQ based on the following equations: LOD = 3.3*σ*/*s* and LOQ = 10*σ*/*s*, where *σ* is standard deviation of intercept and *s* is the slope [39]. Precision was determined by injection of standards at the low, high and mid-point of the calibration curve in triplicate, and the average is reported in Table 1 (standard precision). Intermediate precision was determined by repeating the precision analysis over the duration of the project (3 months). Instrument precision was determined by performing 20 injections from the same standard vial. Matrix-matched precision was determined by the injection of replicate preparations from human serum samples (sample precision). Stock solutions were aliquoted and stored at − 20 °C as recommended by the manufacturer. Solution stability was performed by analysis of the stock standard solution weekly for the first 3 months and then every 4 weeks in order to determine maximum storage duration. Dilute solution were also stored at 2–8 °C in order to access stability when placed in the temperature-controlled autosampler. Dilute solutions were analysed daily as the supplier information indicates a storage stability of 7 days.

Extraction recoveries were determined by obtaining peak area data for calibration standards at low, mid and high calibration curve points. Thyroid hormones were spiked into horse serum both pre- and post-extraction for the low-resolution and pre- and post-dilution for high-resolution LC-MS analyses. To calculate the % recovery, the peak area of the pre-extraction spiked samples was divided by the peak area of the post-extraction spiked samples and multiplied by 100.

## Results and discussion

### Method development

**Fig. 3:**
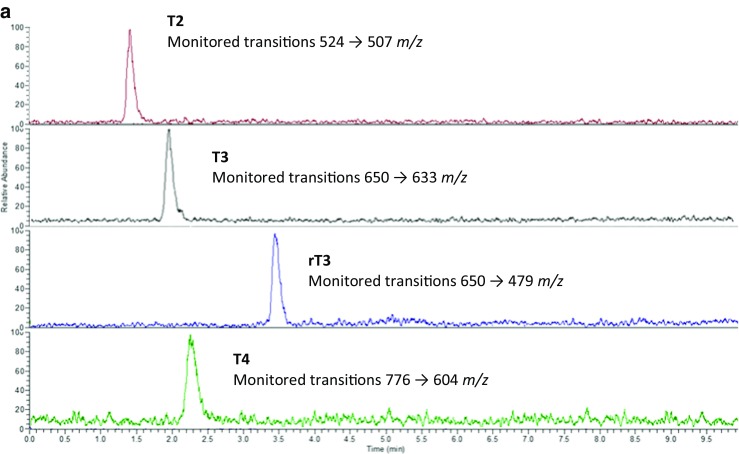

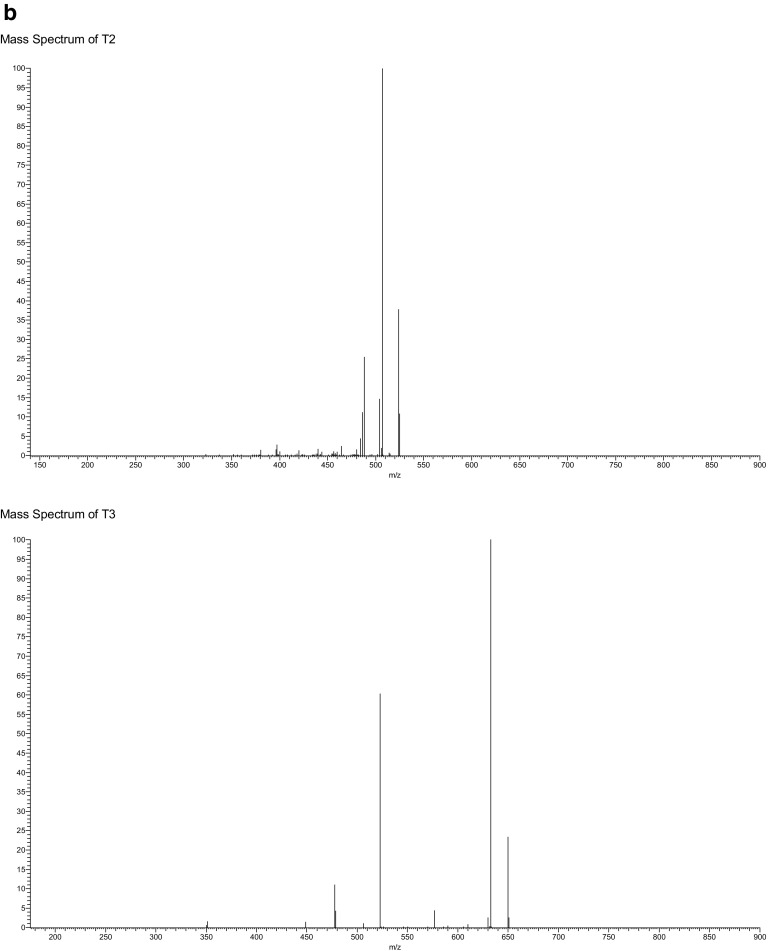

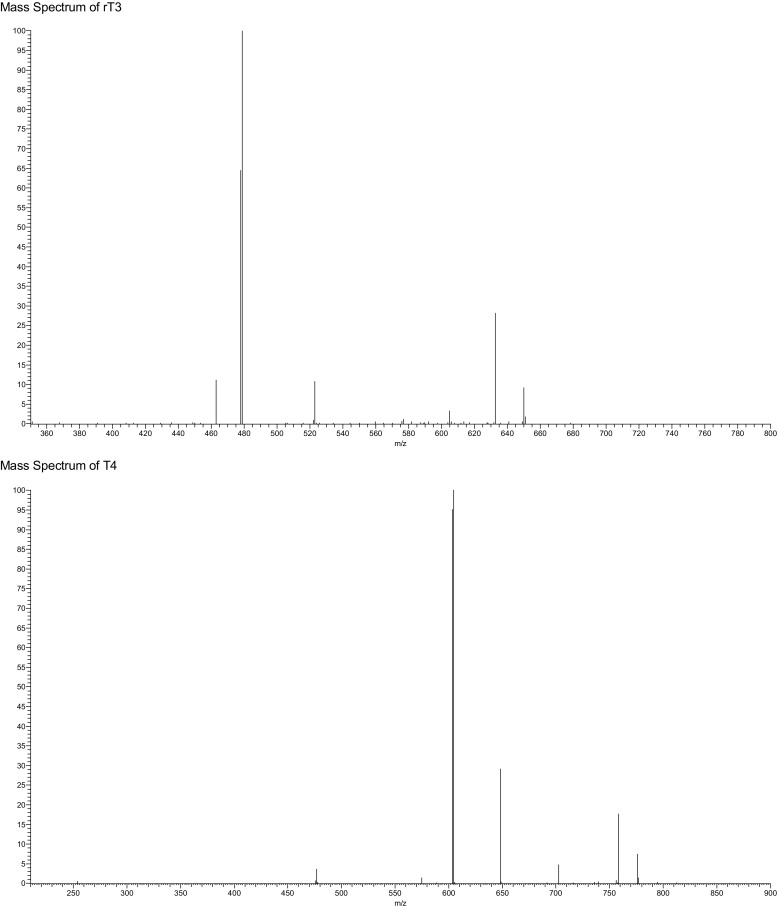
Chromatogram of the separation of “free” thyroid hormones by LC-MS. **a** Horse serum sample. Ions extracted at 507 *m*/*z* (top), 633 *m*/*z* (second), 479 *m*/*z* (third) and 604 *m*/*z* (bottom). **b** Their associated mass spectra

Separation was obtained using a Supelco Ascentis Express™ F5 HPLC column within a run time of 5 min utilising acidified methanol and water as the mobile phase. The ability to separate T3 and rT3 allows for reliable measurement of T3 in serum samples; in order to do this, LC-MS parameters were optimised utilising both the internal tuning parameters and manual gas adjustment in order to achieve the optimum response for the thyroid hormone. MS/MS fragmentation parameters were optimised by direct infusion of each thyroid hormone and the adjustment of parameters until a precursor ion of around 10% relative abundance and two stable product ions were obtained (see for example, Fig. 3b). Horse serum was used to determine specificity and extraction recoveries. Horse blood was chosen as it poses a reduction in the pathogenic risk while still containing the matrix material, of relevance for “free” thyroid hormone determination, which could potentially interfere with the analysis.

The influence of the blood serum matrix was investigated on the recovery of “free” thyroid hormones using both low- and high-resolution LC-MS. Initially, solid phase extraction followed by low-resolution LC-MS sample volume was investigated (Table 2). It was found that if the sample volume was reduced from 1000 to 10 μL, little variation was determined in terms of recoveries from horse blood serum of the four “free” thyroid hormones. Then, subsequently using the 1000 μL horse blood serum sample, the influence of matrix interferences were investigated by running calibration standards for T2, T3, rT3 and T4 in mobile phase and horse blood serum and by spiking the previously extract horse serum with standards at low, mid and high concentration levels. The results of the slopes of their respective calibration graph data are shown in Table 3 and the peak area ratio are shown in Table 4. No matrix effect was established when using solid phase extraction followed by low-resolution LC-MS.

**Table 2:** Method development: investigation of sample volume

Sample volume (mL)	Mean % recovery (*n* = 3) (%RSD)			
	T2	rT3	T3	T4
1	96.0 (3.8)	97.8 (2.5)	98.8 (0.7)	99.2 (0.4)
0.1	97.9 (3.7)	99.0 (3.6)	98.2 (0.6)	99.9 (0.2)
0.01	95.8 (3.8)	97.9 (1.8)	100.4 (0.6)	99.0 (1.5)

**Table 3:** Method development: investigation of matrix effects in horse blood serum using solid phase extraction followed by low-resolution LC-MS for “free” thyroid hormone determination

Compound	Linearity^a^ in mobile phase *y* = *mx* + *c*	*R*^2^ value	Linearity^a^ in blood serum *y* = *mx* + *c*	*R*^2^ value	Matrix effect^b^ (mobile phase: horse serum)
T2	345*x* − 1154	0.9968	339*x* − 1739	0.9972	1.02
T3	335*x* − 1059	0.9951	323*x* − 1250	0.9986	1.04
rT3	332*x* − 1091	0.9962	345*x* − 1136	0.9971	0.96
T4	213*x* − 101	0.9966	209*x* − 29	0.9987	1.02

^a^Concentration range 0–200 pmol/L

^b^Determined using the slope of the calibration graphs in mobile phase and horse blood serum

**Table 4:** Method development: investigation of ion matrix suppression and enhancements in horse blood serum by low-resolution LC-MS for “free” thyroid hormone determination

Compound	Concentration (pmol/L)	Peak area standard	Peak area spiked horse serum	Peak area ratio
T2	10	8016	7712	1.04
	180	35,108	32,340	1.09
	360	67,389	65,856	1.02
T3	10	7854	7382	1.06
	150	30,971	30,171	1.03
	300	68,985	63,705	1.08
rT3	10	8768	8735	1.00
	150	34,351	31,300	1.10
	300	69,290	67,835	1.02
T4	10	8571	8412	1.02
	120	21,483	20,752	1.04
	250	43,987	42,776	1.03

For high-resolution LC-MS, the influence of serial dilution was investigated, based on a 20-pmol/L standard prepared initially in mobile phase and subsequently serially diluted, over five different serial dilutions, in horse blood serum (Table 5). It was found that once the serial dilution equated to a 1 in 100 dilution, no matrix effects were identified. Then, subsequently using the 1 in 100 serial dilution in horse blood serum sample, the influence of matrix interferences were further investigated by running calibration standards for T2, T3, rT3 and T4 in mobile phase and horse blood serum. The results of the slopes of their respective calibration graph data are shown in Table 6. No matrix effect was established when using serial dilution (1 in 100) followed by high-resolution LC-MS.

**Table 5:** Investigation of matrix effects in horse blood serum: influence of dilution factor

Solution	Ratio to 20 pmol/L standard in mobile phase			
	T2	T3	rT3	T4
20 pmol/L standard in mobile phase	1.00	1.00	1.00	1.00
1 in 10 dilution in serum	1.37	2.33	1.37	1.49
1 in 20 dilution in serum	1.26	1.53	1.36	1.45
1 in 40 dilution in serum	1.19	1.23	1.34	1.26
1 in 60 dilution in serum	1.14	1.21	1.17	1.15
1 in 80 dilution in serum	1.10	0.98	1.12	1.08
1 in 100 dilution in serum	1.00	1.00	1.03	1.01

**Table 6:** Investigation of matrix effects in horse blood serum using a serial dilution of 1 in 100 followed by high-resolution LC-MS

Compound	Linearity^a^ in mobile phase *y* = *mx* + *c*	*R*^2^ value	Linearity^a^ in horse blood serum *y* = *mx* + *c*	*R*^2^ value	Matrix effect^b^ (mobile phase: horse blood serum)
T2	210,547*x* − 90,945	0.9992	209,568*x* − 102,529	0.9996	1.00
T3	229,802*x* − 107,024	0.9993	226,213*x* − 99,456	0.9995	1.02
rT3	208,531*x* − 76,627	0.9956	205,189*x* − 126,705	0.9977	1.02
T4	229,796*x* − 109,230	0.9993	229,804*x* − 10,465	0.9993	1.00

^a^Concentration range 0–150 pmol/L

^b^Determined using the slope of the calibration graphs in mobile phase and horse blood serum

Extracted ion chromatograms for the four “free” thyroid hormones in horse serum are shown in Fig. 3 along with their respective mass spectra. No interfering peaks were observed at the same retention time as the compounds of interest, verifying that the method was selective for the detection and quantification of the four thyroid hormones. The calibration curves showed a linear response across the standard range of 0–257, 0–307 and 0–367 pmol/L and 0–129, 0–154 and 0–0183 pmol/L for low-resolution and high-resolution LC-MS, respectively. This was determined by plotting peak area against concentration and an *r*^2^ value of greater than 0.99 was deemed to be a linear response (Table 1). The method also showed good precision for both repeat injections from one vial and replicate injections, with a % RSD of less than 5% for all low, medium and high standard concentrations as well as matrix-matched preparations (Table 1).

**Fig. 4:**
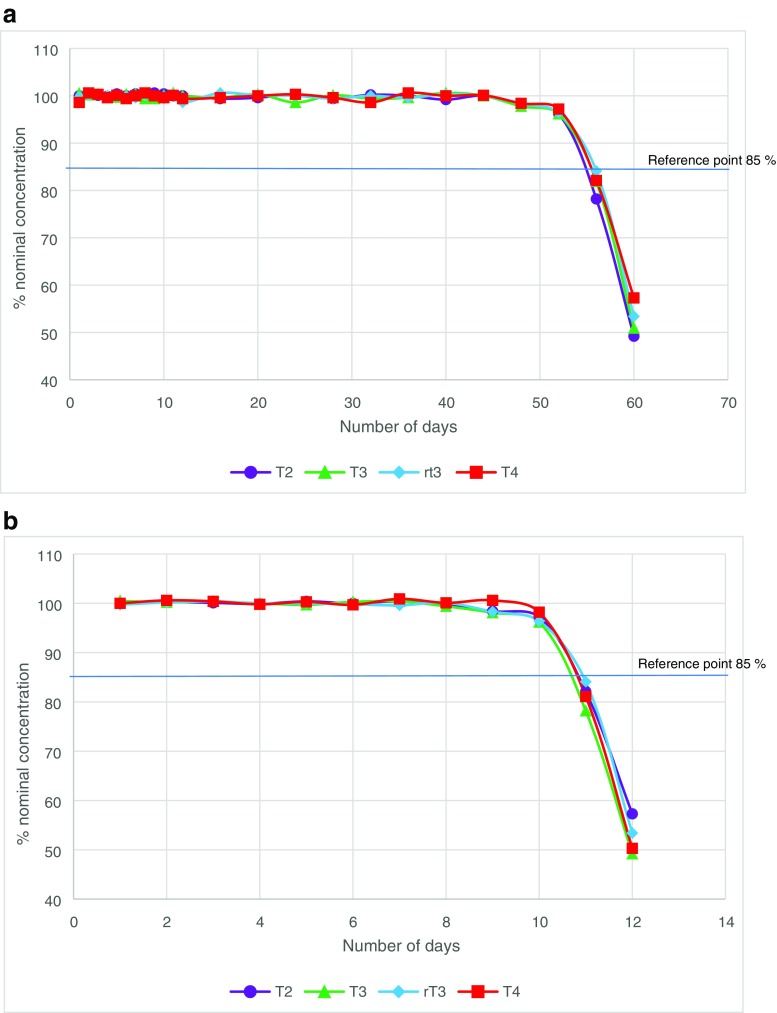
Stock solution stability for solutions stored at **a** − 20 °C and **b** 2–8 °C

Both the low- and high-resolution LC-MS methods showed good sensitivity for all four thyroid hormone with a LOQ for both standards and matrix-matched standards of between 0.17–5.87 and 0.002–0.006 pmol/L for low resolution and high resolution, respectively (Table 1). The improvement in sensitivity for T3 is of importance as it is found at lower concentration within serum samples. In addition, the sensitivity of T3 using ECLIA and ELISA is close to the lower value of the reference interval and therefore causes errors when detecting this compound below the reference range. The sensitivity for both LC-MS methods covers the expected ranges of T3 and T4 and the pre-concentration step included in the SPE method for low-resolution analysis allowed for low levels of thyroid hormones to also be detected. It is also reported in the ECLIA method sheets [17, 18] that there is a permittable error of 30% at the limit of quantification for both T3 and T4 which is not observed in either LC-MS method with a precision of 2.1 and 3.3% for low resolution and 1.8 and 3.9% for high resolution for T4 and T3, respectively. The extraction recoveries for the SPE extraction method were assessed by spiking a known standard concentration into horse blood serum samples and then extracting as per the sample preparation method for low-resolution LC-MS. The extraction method was assessed at low and high levels of thyroid hormones to ensure consistence across a range of sample concentrations. The recoveries were 96.4% and 98.5% for the low concentration and high concentration samples, respectively. Stock solution stability was accessed for all four thyroid hormones until such time that the concentration obtained differed by ± 15% of the nominal concentration. It was found that the stock solutions were stable at − 20 °C for 52 weeks. The dilute solutions were stable at 2–8 °C for 10 days ensuring stability of solutions during analysis (Fig. 4).

### Direct comparison of analytical methods using hospital-derived blood serum samples

**Fig. 5:**
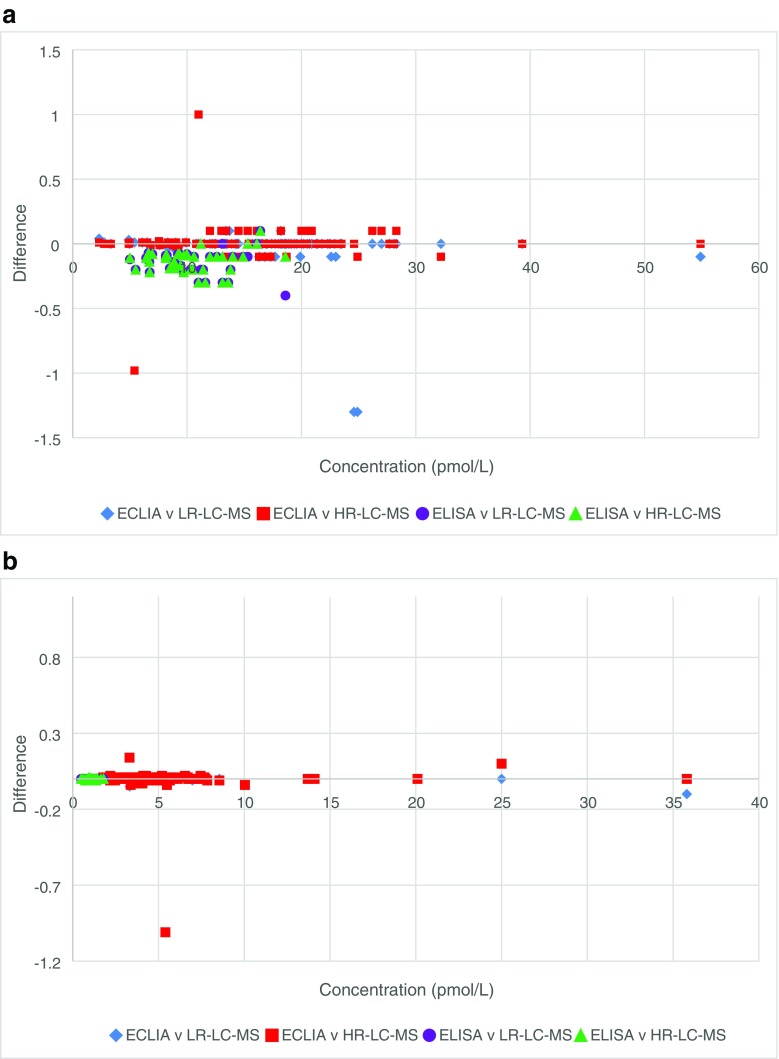
Measurement agreement plots for **a** T4 by ELISA or ECLIA versus LC-MS and **b** T3 by ELISA or ECLIA versus LC-MS. ECLIA, *N* = 118; ELISA, *N* = 40; LR-LC-MS, *N* = 158; HR-LC-MS, *N* = 158

Human serum samples were provided by the Royal Victoria Infirmary which had been previously tested for T4 and T3 and were analysed, in a blind study, using the validated LC-MS methods for both low-resolution and high-resolution LC-MS (Electronic Supplementary Material (ESM) Table S1). The analysis showed that the method for both low resolution and high resolution were comparable to the clinical routine testing methods of ECLIA and the more readily available ELISA. Figure 5 shows the measurement agreement analysis of each method compared to the results obtained by LC-MS. The null hypothesis for the statistical analysis is that the means of the comparable methods for each hormone are the same. In order to accept the null hypothesis at the 95% confidence interval, a *p* value of > 0.05 should be obtained. Statistical paired sample *t* test using SPSS found that there was no statistical different between the means of each set of data at the 95% confidence interval (Table 7). However, it was not possible to obtain mean data for the ECLIA data as only a single clinical measurement was made.

**Table 7:** Statistical *p* values between low and high-resolution LC-MS and an immunoassay method

Compound: technique	Paired sample *t* test	
	All samples	Sample concentration < 10 pmol/L
T4: ELISA v LR-LC-MS	0.069	0.330
T3: ELISA v LR-LC-MS	0.074	0.347
T4: ELISA v HR-LC-MS	0.137	0.334
T3: ELISA v HR-LC-MS	0.140	0.326

*ELISA* enzyme-linked immunosorbent assay (*N* = 40), *LR-LC-MS* low-resolution liquid chromatography-mass spectrometry (*N* = 158), *HR-LC-MS* high-resolution liquid chromatography-mass spectrometry (*N* = 158)

A comparison of all four methods (Table 8) shows a significant reduction in analysis time for high-resolution LC-MS and also batch analysis by low-resolution LC-MS from 20 to 7 and 8.75 min, respectively. Both LC-MS method are also able to analyse all four hormones in one run, meaning that repeat tests do not need to be performed for each hormone. However, due to the SPE extraction used for low-resolution LC-MS, a higher level of operator skill is required when compared with the other three methods, which mainly require the operator to be skilled in basic laboratory techniques and the use of specialist software. The increase in skill level and training requirements may still be a more favourable option over the high-resolution LC-MS for some laboratories due to the increase in capital cost associated with the high-resolution instrumentation.

**Table 8:** Comparison of current and new methodologies

	ECLIA	ELISA	Low-resolution LC-MS	High-resolution LC-MS
Number of thyroid hormones analysed per run	1	1	4	4
Sample preparation technique	Pipetting/dilution	Pipetting/dilution	SPE	Pipetting/dilution
Operator skill	Medium	Low–medium	Medium to high	Medium
Automation	Yes	No	No	Yes
Sample preparation time, per sample (min)	2	2	Batch analysis 3.75; individual analysis 30	2
Analysis time, per sample (min)	18	180^a^	5	5
Total time (min)	20	182	Batch analysis 8.75; individual analysis 35	7
Approximate capital cost	Medium	Low	Medium	High
Typical cost per run	Low	High	Low	Low

Operator skill scale: low, basic laboratory skills; medium, use of specialised software; high, use of specialised laboratory skill

^a^96-well plate capable of analysing 16 samples in triplicate

The developed method reduces the run time from 14 to 5 min and as an isocratic method it removes the need for column re-equilibration in between samples. The method also shows improvements over those previously published in terms of precision, extraction recoveries and correlation [32–35]. Tanoue et al. [35] reported extraction recoveries, for low concentration, 82.9, 81.9, 83.5 and 62.7% and for high concentration, 86.9, 90.5, 90.0 and 69.6% for T4, T3, rT3 and T2, respectively. They also reported [35] precision data with typical %CV ranging between 6.1 and 8.5% for T4 and 3.8 and 8.6% for T3. In this work, we report significantly higher extraction recoveries of 97.9, 95.9, 98.2 and 99.7%, at low concentration, and 97.9, 95.8, 99.3 and 99.0%, at high concentration, for T4, T3, rT3 and T2, respectively. Precision also showed improvements with %CVs of between 2.2–3.3 and 1.8–3.8 for low-resolution and high-resolution LC-MS instruments, respectively (Table 1). Data previously published [32–35] showed limited correlation between LC-MS and immunoassay methods. Typical reported correlation coefficients of 0.67, 0.74 and 0.90 for T3 only at low, medium and high concentrations, respectively [34]; 0.80 and 0.48 for T4 only in samples from cats and dogs, respectively [35]; 0.37 and 0.53 for T3 and T4, respectively [32]; and 0.30 and 0.55 for T3 and T4, respectively [33]. This newly reported method produced data which showed good correlation (all > 0.99) compared to the currently used immunoassay methods both across the full concentration range and when focusing on low level samples (Fig. 5).

## Conclusion

LC-MS methods for the analysis of “free” thyroid hormones have been previously published; however, these have shown a large variation in sample extraction recoveries and precision data. Therefore, offering limited improvements on the currently used ECLIA method. These papers also have limited comparison of new with existing established methodologies and those which have included comparison show limited correlation between the methods.

The developed LC-MS methods are able to identify and quantify the four “free” thyroid hormones with improved sensitivity and accuracy for T3 and T4 compared to the methods currently used within the NHS and research facilities. The developed LC-MS methods showed no statistical difference when compared to the immunoassay methods. The LC-MS methods have the added advantage of being able to analyse all four “free” thyroid hormones within one instrument run as opposed to the need to run each thyroid hormone separately when using both ECLIA and ELISA, therefore significantly reducing the analysis time for both the low- and high-resolution LC-MS methods. Although not tested as part of this study, automation could also be used by utilising autosampler programing to perform sample dilution on the high-resolution LC-MS method and the possibility of in-line SPE for the low-resolution LC-MS method.

The high-resolution LC-MS method shows the greatest improvement in terms of sensitivity and analysis time; however, due to the increase in capital cost, this may be less favourable in certain laboratories. Therefore the low-resolution LC-MS method would be a useful compromise as the increase in sensitivity and reduction in analysis time would still be achievable but with a reduced capital expenditure. With both LC-MS methods, there would be a requirement for staff training in the form of software training and/or SPE training which would need to be factored into expenditure costs.

## Electronic supplementary material

ESM 1 (PDF 319 kb)

## References

[1] Sinha RA, Singh BK, Yen PM (2014). Thyroid hormone regulation of hepatic lipid and carbohydrate metabolism. Trends Endocrinol Metab.

[2] Goodman HM (2009). Basic medical endocrinology.

[3] Gu J, Soldin OP, Soldin SJ (2007). Simultaneous quantification of free triiodothyronine and free thyroxine by isotope dilution tandem mass spectrometry. Clin Biochem.

[4] Kapelari K, Kirchlechner C, Hogler W, Schweitzer K, Virgolini I, Moncayo R (2008). Pediatric reference intervals for thyroid hormone levels from birth to adulthood: a retrospective study. BMC Endocr Disord.

[5] Lem AJ, de Rijke YB, van Toor H, de Ridder MA, Visser TJ, Hokken-Koelega AC (2012). Serum thyroid hormone levels in healthy children from birth to adulthood and in short children born small for gestational age. J Clin Endocrinol Metab.

[6] Kvetny J (2003). The significance of clinical euthyroidism on reference range for thyroid hormones. Eur J Intern Med.

[7] NHS TNuTH. Free T3, serum 2018 [Available from: https://secure.newcastlelaboratories.com/test-directory/test/free-t3-serum/.

[8] NHS TNuTH. Free T4, serum 2018 [Available from: https://secure.newcastlelaboratories.com/test-directory/test/free-t4-serum/.

[9] NHS. Hyperthyroidism 2018 [Available from: https://www.nhs.uk/conditions/overactive-thyroid-hyperthyroidism/diagnosis/.

[10] Chaker L, Bianco AC, Jonklaas J, Peeters RP. Hypothyroidism. Lancet. 2017. 10.1016/S0140-6736(17)30703-1 PMC6619426 28336049

[11] Dunn D, Turner C (2016). Hypothyroidism in women. Nurs Womens Health.

[12] Gilbert J (2017). Hypothyroidism. Medicine..

[13] NHS. Hypothyroidism 2018 [Available from: https://www.nhs.uk/conditions/underactive-thyroid-hypothyroidism/.

[14] Sanchez-Carbayo M, Mauri M, Alfayate R, Miralles C, Soria F (1999). Analytical and clinical evaluation of TSH and thyroid hormones by electrochemiluminescent immunoassays. Clin Biochem.

[15] Soldin OP, Hilakivi-Clarke L, Weiderpass E, Soldin SJ (2004). Trimester-specific reference intervals for thyroxine and triiodothyronine in pregnancy in iodine-sufficient women using isotope dilution tandem mass spectrometry and immunoassays. Clin Chim Acta.

[16] Kazerouni F, Amirrasouli H (2012). Performance characteristics of three automated immunoassays for thyroid hormones. Capsian J Intern Med.

[17] Welsh KJ, Soldin SJ (2016). How reliable are free thyroid and total T3 hormone assays?. Eur J Endocrinol.

[18] Cobas. FT3 III method sheet 2017 [Available from: http://labogids.sintmaria.be/sites/default/files/files/ft3_iii_2017-03_v2.pdf.

[19] Cobas. FT4 II method sheet 2017 [Available from: http://labogids.sintmaria.be/sites/default/files/files/ft4_ii_2013-05_v2.pdf.

[20] Scientific T. TriiodothyronineT3 competitive ELISA method [Available from: https://www.thermofisher.com/order/catalog/product/EIAT3C.

[21] Scientific T. Thyroxine T4 competitive ELISA method [Available from: https://www.thermofisher.com/order/catalog/product/EIAT4C?SID=srch-srp-EIAT4C.

[22] Jonklaas J, Sathasivam A, Wang H, Gu J, Burman KD, Soldin SJ (2014). Total and free thyroxine and triiodothyronine: measurement discrepancies, particularly in inpatients. Clin Biochem.

[23] Wu AH, French D (2013). Implementation of liquid chromatography/mass spectrometry into the clinical laboratory. Clin Chim Acta.

[24] Yong S, Chen Y, Lee TK, Lee HK (2014). Determination of total thyroxine in human serum by hollow fiber liquid-phase microextraction and liquid chromatography-tandem mass spectrometry. Talanta..

[25] Zhang Y, Conrad AH, Conrad GW (2005). Detection and quantification of 3,5,3′-triiodothyronine and 3,3′,5′-triiodothyronine by electrospray ionization tandem mass spectrometry. J Am Soc Mass Spectrom.

[26] Couldwell AM, Thomas MC, Mitchell TW, Hulbert AJ, Blanksby SJ (2005). Tandem mass spectrometry of deprotonated iodothyronines. Rapid Commun Mass Sp.

[27] Grebenstein N, Frank J (2012). Rapid baseline-separation of all eight tocopherols and tocotrienols by reversed-phase liquid-chromatography with a solid-core pentafluorophenyl column and their sensitive quantification in plasma and liver. J Chromatogr A.

[28] Lemasson E, Bertin S, Hennig P, Lesellier E, West C (2018). Impurity profiling of drug candidates: analytical strategies using reversed-phase and mixed-mode high-performance liquid chromatography methods. J Chromatogr A.

[29] Smidova B, Satinsky D, Dostalova K, Solich P (2017). The pentafluorophenyl stationary phase shows a unique separation efficiency for performing fast chromatography determination of highbush blueberry anthocyanins. Talanta..

[30] Fibigr J, Satinsky D, Solich P (2017). A new approach to the rapid separation of isomeric compounds in a Silybum marianum extract using UHPLC core-shell column with F5 stationary phase. J Pharmaceut Biomed.

[31] Yang M, Fazio S, Munch D, Drumm P (2005). Impact of methanol and acetonitrile on separations based on pi-pi interactions with a reversed-phase phenyl column. J Chromatogr A.

[32] Welsh KJ, Stolze BR, Yu X, Podsiadlo TR, Kim LS, Soldin SJ (2017). Assessment of thyroid function in intensive care unit patients by liquid chromatography tandem mass spectrometry methods. Clin Biochem.

[33] Gounden V, Jonklaas J, Soldin SJ (2014). A pilot study: subclinical hypothyroidism and free thyroid hormone measurement by immunoassay and mass spectrometry. Clin Chim Acta.

[34] Masika LS, Zhao Z, Soldin SJ (2016). Is measurement of TT3 by immunoassay reliable at low concentrations? A comparison of the Roche Cobas 6000 vs. LC-MSMS. Clin Biochem.

[35] Tanoue R, Kume I, Yamamoto Y, Takaguchi K, Nomiyama K, Tanabe S (2018). Determination of free thyroid hormones in animal serum/plasma using ultrafiltration in combination with ultra-fast liquid chromatography-tandem mass spectrometry. J Chromatogr A.

[36] European Medicines Agency guideline bioanalytical method validation. 2011. 10.4155/bio.12.44 22533559

[37] Messer NT, Riddle WT, Traub-Dargatz JL, Dargantz DA, Refsal KJ, Thompson DL (1998). Thyroid hormone levels in thoroughbred mares and their foals at parturition. American Association of Equine Practitioners.

[38] ICH guideline validation of analytical procedures—test and methodology [Available from: http://www.ich.org/products/guidelines/quality/quality-single/article/validation-of-analytical-procedures-text-and-methodology.html.

