# Supplementary material for: A direct comparison of liquid chromatography-mass spectrometry with clinical routine testing immunoassay methods for the detection and quantification of thyroid hormones in blood serum
Source: Anal Bioanal Chem. 2019 May 11;411(13):2839–53. doi: 10.1007/s00216-019-01724-2 (PMC6522465; doi:10.1007/s00216-019-01724-2)
Supplement: Supplementary file 1 — (PDF 319 kb) [file 216_2019_1724_MOESM1_ESM.pdf]

Analytical and Bioanalytical Chemistry

Electronic Supplementary Material

A direct comparison of liquid chromatography-mass spectrometry with clinical routine testing immunoassay methods for the detection and quantification of thyroid hormones in blood serum

Samantha L. Bowerbank, Michelle G. Carlin, John R. Dean

Table S1 Sample results

Sample ID	T2 Concentration (pmol/L)		T3 Concentration (pmol/L)			T4 Concentration (pmol/L)			rT3 Concentration (pmol/L)	
	LR LC-MS (n = 3)	HR LC-MS (n = 3)	ECLIA	LR LC-MS (n = 3)	HR LC-MS (n = 3)	ECLIA	LR LC-MS (n = 3)	HR LC-MS (n = 3)	LR LC-MS (n = 3)	HR LC-MS (n = 3)
#1	13.9 ± 0.08	13.9 ± 0.09	1.10	1.10 ± 0.06	1.10 ± 0.05	2.31	2.35 ± 0.16	2.32 ± 0.07	1.17 ± 0.05	1.19 ± 0.02
#2	13.7 ± 0.02	13.7 ± 0.09	NT	NT	NT	2.75	2.76 ± 0.08	2.75 ± 0.01	1.43 ± 0.07	1.46 ± 0.02
#3	20.9 ± 0.07	20.8 ± 0.03	2.18	2.18 ± 0.02	2.17 ± 0.03	3.33	3.33 ± 0.13	3.33 ± 0.05	1.34 ± 0.07	1.37 ± 0.02
#4	11.9 ± 0.05	11.9 ± 0.04	NT	NT	NT	4.90	4.93 ± 0.20	4.90 ± 0.03	1.02 ± 0.05	1.04 ± 0.01
#5	11.4 ± 0.02	11.3 ± 0.07	NT	NT	NT	4.96	4.96 ± 0.17	4.97 ± 0.01	0.65 ± 0.02	0.66 ± 0.05
#6	12.9 ± 0.07	12.9 ± 0.08	2.63	2.63 ± 0.07	2.63 ± 0.01	5.40	5.41 ± 0.07	4.42 ± 0.11	0.94 ± 0.05	0.96 ± 0.01
#7	16.3 ± 0.02	16.2 ± 0.01	NT	NT	NT	6.08	6.09 ± 0.14	6.09 ± 0.02	1.27 ± 0.05	1.29 ± 0.02
#8	14.2 ± 0.08	14.1 ± 0.02	3.50	3.50 ± 0.02	3.51 ± 0.04	6.51	6.52 ± 0.31	6.52 ± 0.01	0.96 ± 0.04	0.98 ± 0.07
#9	13.2 ± 0.03	13.2 ± 0.08	NT	NT	NT	6.80	6.78 ± 0.26	6.79 ± 0.02	1.19 ± 0.05	1.21 ± 0.08
#10	63.6 ± 0.08	63.5 ± 0.04	NT	NT	NT	7.54	7.55 ± 0.10	7.56 ± 0.02	1.39 ± 0.06	1.42 ± 0.01
#11	12.9 ± 0.02	12.8 ± 0.08	2.46	2.46 ± 0.06	2.45 ± 0.04	7.60	7.59 ± 0.16	7.59 ± 0.02	0.95 ± 0.06	0.97 ± 0.06
#12	16.1 ± 0.02	16.1 ± 0.07	NT	NT	NT	8.31	8.28 ± 0.09	8.31 ± 0.02	1.10 ± 0.05	1.12 ± 0.07
#13	12.9 ± 0.02	12.9 ± 0.09	NT	NT	NT	8.31	8.32 ± 0.04	8.32 ± 0.03	0.99 ± 0.04	1.01 ± 0.06
#14	11.2 ± 0.01	11.2 ± 0.08	3.88	3.88 ± 0.02	3.89 ± 0.02	8.78	8.75 ± 0.21	8.77 ± 0.02	0.83 ± 0.03	0.84 ± 0.03
#15	14.7 ± 0.09	14.7 ± 0.03	NT	NT	NT	8.88	8.88 ± 0.17	8.88 ± 0.06	1.44 ± 0.07	1.47 ± 0.04
#16	12.8 ± 0.06	12.8 ± 0.07	NT	NT	NT	8.90	8.88 ± 0.10	8.90 ± 0.08	0.78 ± 0.13	0.79 ± 0.05
#17	5.32 ± 0.07	5.29 ± 0.09	NT	NT	NT	9.00	9.01 ± 0.24	9.01 ± 0.01	1.51 ± 0.06	1.54 ± 0.01
#18	10.8 ± 0.04	10.7 ± 0.09	2.48	2.48 ± 0.06	2.48 ± 0.11	9.04	9.04 ± 0.22	9.05 ± 0.03	1.44 ± 0.07	1.47 ± 0.02
#19	14.9 ± 0.09	14.8 ± 0.03	NT	NT	NT	9.10	9.07 ± 0.13	9.08 ± 0.02	1.53 ± 0.08	1.56 ± 0.09
#20	13.4 ± 0.07	13.4 ± 0.01	NT	NT	NT	9.23	9.20 ± 0.08	9.20 ± 0.03	1.39 ± 0.07	1.42 ± 0.09
#21	20.7 ± 0.07	20.6 ± 0.11	3.77	3.77 ± 0.08	3.77 ± 0.06	9.90	9.91 ± 0.34	9.91 ± 0.04	1.82 ± 0.08	1.85 ± 0.01
#22	16.5 ± 0.02	16.4 ± 0.07	2.20	2.20 ± 0.02	2.21 ± 0.04	10.8	10.8 ± 0.04	10.8 ± 0.21	1.16 ± 0.05	1.18 ± 0.07
#23	17.7 ± 0.03	17.7 ± 0.09	4.17	4.17 ± 0.04	4.18 ± 0.01	10.8	10.8 ± 0.08	10.8 ± 0.06	1.34 ± 0.07	1.37 ± 0.08
#24	11.2 ± 0.14	11.1 ± 0.07	3.32	3.27 ± 0.03	3.33 ± 0.04	11.0	11.0 ± 0.08	12.0 ± 0.03	0.62 ± 0.03	0.63 ± 0.04
#25	14.4 ± 0.08	14.3 ± 0.01	2.78	2.78 ± 0.02	2.78 ± 0.03	11.5	11.5 ± 0.14	11.5 ± 0.15	1.16 ± 0.05	1.18 ± 0.07

#26	11.3 ± 0.04	11.3 ± 0.08	3.04	3.04 ± 0.01	3.05 ± 0.09	12.0	12.0 ± 0.15	12.1 ± 0.02	0.75 ± 0.03	0.76 ± 0.05
#27	15.7 ± 0.02	15.7 ± 0.08	6.98	6.97 ± 0.04	6.98 ± 0.04	12.2	12.2 ± 0.25	12.2 ± 0.01	0.95 ± 0.05	0.97 ± 0.06
#28	12.5 ± 0.06	12.4 ± 0.06	4.98	4.98 ± 0.02	4.98 ± 0.13	12.4	12.4 ± 0.15	12.4 ± 0.10	1.09 ± 0.05	1.11 ± 0.07
#29	11.8 ± 0.05	11.8 ± 0.09	4.75	4.75 ± 0.02	4.76 ± 0.05	12.5	12.5 ± 0.06	12.5 ± 0.03	0.69 ± 0.04	0.71 ± 0.05
#30	12.2 ± 0.01	12.1 ± 0.07	4.48	4.48 ± 0.02	4.47 ± 0.02	13.0	13.0 ± 0.20	13.1 ± 0.01	0.85 ± 0.04	0.87 ± 0.06
#31	13.9 ± 0.06	13.8 ± 0.06	4.99	4.99 ± 0.02	4.99 ± 0.01	13.3	13.3 ± 0.19	13.3 ± 0.04	1.00 ± 0.05	1.02 ± 0.07
#32	11.7 ± 0.08	11.6 ± 0.07	3.90	3.90 ± 0.01	3.91 ± 0.01	13.4	13.4 ± 0.04	13.4 ± 0.14	0.79 ± 0.03	0.81 ± 0.05
#33	8.87 ± 0.11	8.82 ± 0.09	4.09	4.09 ± 0.02	4.09 ± 0.03	13.4	13.5 ± 0.16	13.5 ± 0.02	0.64 ± 0.03	0.65 ± 0.05
#34	10.7 ± 0.04	10.7 ± 0.03	4.00	4.00 ± 0.03	3.99 ± 0.07	13.5	13.5 ± 0.11	13.4 ± 0.03	0.72 ± 0.07	0.73 ± 0.07
#35	45.3 ± 0.05	45.3 ± 0.02	4.53	4.53 ± 0.01	4.53 ± 0.02	13.7	13.8 ± 0.35	13.7 ± 0.02	1.59 ± 0.04	1.62 ± 0.05
#36	48.3 ± 0.02	48.2 ± 0.01	4.11	4.11 ± 0.01	4.11 ± 0.21	13.9	13.9 ± 0.16	13.9 ± 0.06	1.11 ± 0.07	1.13 ± 0.04
#37	36.1 ± 0.06	36.0 ± 0.02	4.52	4.52 ± 0.07	4.51 ± 0.04	14.2	14.2 ± 0.12	14.2 ± 0.03	1.27 ± 0.05	1.30 ± 0.08
#38	19.6 ± 0.06	20.0 ± 0.09	3.19	3.19 ± 0.03	3.19 ± 0.02	14.3	14.3 ± 0.24	14.2 ± 0.05	3.89 ± 0.09	3.87 ± 0.05
#39	32.9 ± 0.08	33.0 ± 0.08	4.70	4.70 ± 0.02	4.70 ± 0.01	14.4	14.4 ± 0.07	14.3 ± 0.01	6.62 ± 0.08	6.59 ± 0.08
#40	14.4 ± 0.13	14.4 ± 0.04	4.91	4.91 ± 0.03	4.91 ± 0.02	14.5	14.5 ± 0.41	14.6 ± 0.05	6.30 ± 0.08	6.27 ± 0.08
#41	11.8 ± 0.02	12.0 ± 0.06	4.66	4.65 ± 0.02	4.65 ± 0.02	15.3	15.3 ± 0.16	15.4 ± 0.02	<LOQ	<LOQ
#42	6.32 ± 0.04	6.34 ± 0.11	4.83	4.83 ± 0.04	4.83 ± 0.06	15.4	15.4 ± 0.20	15.4 ± 0.01	1.30 ± 0.12	1.29 ± 0.02
#43	9.90 ± 0.06	9.91 ± 0.02	3.71	3.71 ± 0.07	3.72 ± 0.04	15.4	15.4 ± 0.08	15.4 ± 0.03	1.11 ± 0.06	1.10 ± 0.01
#44	17.1 ± 0.16	17.1 ± 0.08	7.16	7.16 ± 0.02	7.16 ± 0.08	15.7	15.7 ± 0.38	15.7 ± 0.09	3.78 ± 0.06	3.76 ± 0.05
#45	18.1 ± 0.08	18.2 ± 0.04	5.26	5.26 ± 0.13	5.25 ± 0.02	16.0	16.0 ± 0.28	16.0 ± 0.02	2.97 ± 0.04	2.96 ± 0.04
#46	27.3 ± 0.03	27.3 ± 0.01	5.23	5.23 ± 0.08	5.23 ± 0.05	16.1	16.1 ± 0.15	16.2 ± 0.01	6.23 ± 0.10	6.20 ± 0.08
#47	12.1 ± 0.09	12.1 ± 0.04	4.81	4.81 ± 0.03	4.81 ± 0.03	16.3	16.2 ± 0.26	16.2 ± 0.03	3.75 ± 0.24	3.73 ± 0.04
#48	21.4 ± 0.10	21.4 ± 0.09	6.06	6.06 ± 0.01	6.07 ± 0.05	16.3	16.3 ± 0.15	16.4 ± 0.02	3.99 ± 0.06	3.97 ± 0.05
#49	17.2 ± 0.06	17.3 ± 0.13	5.70	5.70 ± 0.01	5.71 ± 0.04	16.3	16.3 ± 0.09	16.3 ± 0.02	<LOQ	<LOQ
#50	22.0 ± 0.03	22.0 ± 0.07	6.73	6.73 ± 0.08	6.73 ± 0.10	16.5	16.5 ± 0.10	16.5 ± 0.01	5.70 ± 0.07	5.68 ± 0.07
#51	14.3 ± 0.08	14.2 ± 0.02	5.40	5.40 ± 0.01	5.40 ± 0.01	16.6	16.6 ± 0.16	16.5 ± 0.01	2.35 ± 0.05	2.33 ± 0.03
#52	19.5 ± 0.04	19.5 ± 0.03	3.93	3.93 ± 0.04	3.92 ± 0.01	16.8	16.7 ± 0.26	16.8 ± 0.03	<LOQ	<LOQ
#53	40.7 ± 0.08	40.5 ± 0.02	5.21	5.21 ± 0.17	5.21 ± 0.06	16.9	16.9 ± 0.22	16.9 ± 0.05	<LOQ	<LOQ
#54	44.3 ± 0.06	44.3 ± 0.04	4.65	4.65 ± 0.02	4.65 ± 0.03	17.0	17.0 ± 0.33	17.0 ± 0.01	9.82 ± 0.14	9.77 ± 0.13
#55	35.7 ± 0.03	35.7 ± 0.02	1.78	1.78 ± 0.03	1.79 ± 0.04	17.3	17.2 ± 0.07	17.2 ± 0.03	8.24 ± 0.12	8.20 ± 0.11

#56	36.6 ± 0.17	36.6 ± 0.08	5.43	5.43 ± 0.01	5.43 ± 0.09	17.4	17.4 ± 0.05	17.4 ± 0.01	<LOQ	<LOQ
#57	35.4 ± 0.04	35.4 ± 0.01	4.13	4.13 ± 0.01	4.13 ± 0.02	17.7	17.6 ± 0.12	17.7 ± 0.03	8.56 ± 0.12	8.52 ± 0.11
#58	50.7 ± 0.06	50.8 ± 0.09	7.84	7.83 ± 0.02	7.83 ± 0.01	17.7	17.7 ± 0.08	17.7 ± 0.01	1.33 ± 0.19	1.32 ± 0.17
#59	28.2 ± 0.09	28.2 ± 0.12	5.25	5.25 ± 0.04	5.26 ± 0.07	18.0	18.0 ± 0.14	18.0 ± 0.01	5.88 ± 0.09	5.85 ± 0.08
#60	27.5 ± 0.02	27.6 ± 0.08	3.38	3.38 ± 0.02	3.34 ± 0.02	18.2	18.2 ± 0.23	18.2 ± 0.04	<LOQ	<LOQ
#61	13.2 ± 0.10	13.4 ± 0.04	4.99	4.99 ± 0.02	4.99 ± 0.04	18.2	18.3 ± 0.12	18.3 ± 0.02	2.04 ± 0.03	2.03 ± 0.03
#62	18.4 ± 0.06	18.3 ± 0.10	4.27	4.27 ± 0.02	4.29 ± 0.01	18.3	18.3 ± 0.36	18.3 ± 0.01	3.33 ± 0.06	3.31 ± 0.04
#63	12.1 ± 0.03	12.3 ± 0.05	3.31	3.31 ± 0.02	3.45 ± 0.01	18.3	18.3 ± 0.10	18.3 ± 0.01	2.23 ± 0.02	2.22 ± 0.03
#64	21.9 ± 0.12	22.0 ± 0.08	6.12	6.12 ± 0.01	6.12 ± 0.02	18.5	18.5 ± 0.15	18.5 ± 0.02	2.54 ± 0.03	2.53 ± 0.03
#65	15.9 ± 0.03	16.2 ± 0.07	2.19	2.19 ± 0.07	2.21 ± 0.01	18.5	18.5 ± 0.07	18.5 ± 0.05	4.59 ± 0.08	4.56 ± 0.06
#66	7.97 ± 0.14	8.93 ± 0.06	3.33	3.33 ± 0.02	3.33 ± 0.08	18.6	18.5 ± 0.16	18.6 ± 0.02	2.26 ± 0.04	2.25 ± 0.03
#67	12.5 ± 0.04	12.5 ± 0.07	3.89	3.89 ± 0.03	3.90 ± 0.03	18.7	18.7 ± 0.10	18.6 ± 0.02	0.83 ± 0.20	0.82 ± 0.01
#68	25.5 ± 0.05	25.5 ± 0.07	4.48	4.48 ± 0.02	4.48 ± 0.14	18.9	18.9 ± 0.06	18.9 ± 0.07	4.31 ± 0.07	4.29 ± 0.06
#69	9.08 ± 0.02	9.06 ± 0.12	3.68	3.68 ± 0.07	3.66 ±0.02	19.1	19.1 ± 0.02	19.1 ± 0.01	2.45 ± 0.03	2.44 ± 0.03
#70	9.98 ± 0.10	10.1 ± 0.07	4.77	4.77 ± 0.03	4.77 ± 0.06	19.1	19.1 ± 0.01	19.1 ± 0.02	1.57 ± 0.02	1.56 ± 0.02
#71	10.6 ± 0.06	10.8 ± 0.10	5.67	5.67 ± 0.01	5.66 ± 0.01	19.4	19.4 ± 0.03	19.4 ± 0.01	1.49 ± 0.03	1.48 ± 0.02
#72	18.4 ± 0.05	18.4 ± 0.05	4.83	4.83 ± 0.01	4.82 ± 0.04	19.6	19.6 ± 0.02	19.6 ± 0.01	1.31 ± 0.13	1.30 ± 0.04
#73	11.1 ± 0.09	11.1 ± 0.05	6.54	6.54 ± 0.03	6.56 ± 0.03	19.9	19.8 ± 0.04	19.9 ± 0.02	1.53 ± 0.03	1.52 ± 0.02
#74	14.9 ± 0.08	15.2 ± 0.07	4.05	4.05 ± 0.02	4.05 ± 0.02	20.0	20.0 ± 0.01	20.1 ± 0.04	2.24 ± 0.03	2.23 ± 0.03
#75	7.74 ± 0.09	7.78 ± 0.04	5.25	5.25 ± 0.02	5.25 ± 0.02	20.2	20.2 ± 0.02	20.2 ± 0.02	1.60 ± 0.04	1.60 ± 0.04
#76	7.64 ± 0.12	7.67 ± 0.04	6.70	6.70 ± 0.02	6.71 ± 0.06	20.5	20.5 ± 0.03	20.5 ± 0.02	1.96 ± 0.05	1.96 ± 0.07
#77	11.6 ± 0.10	11.6 ± 0.05	4.60	4.60 ± 0.04	4.61 ± 0.04	20.7	20.7 ± 0.02	20.8 ± 0.01	1.84 ± 0.04	1.86 ± 0.04
#78	6.64 ± 0.09	6.67 ± 0.06	2.15	2.15 ± 0.04	2.15 ± 0.05	20.9	20.9 ± 0.01	21.0 ± 0.01	1.40 ± 0.06	1.40 ± 0.03
#79	6.32 ± 0.12	6.35 ± 0.05	5.40	5.40 ± 0.04	4.39 ± 0.07	21.3	21.3 ± 0.03	21.3 ± 0.01	0.89 ± 0.03	0.90 ± 0.04
#80	7.19 ± 0.09	7.22 ± 0.05	5.51	5.51 ± 0.03	5.52 ± 0.21	21.4	21.4 ± 0.03	21.4 ± 0.02	1.29 ± 0.02	1.30 ± 0.04
#81	9.04 ± 0.08	9.08 ± 0.03	5.49	5.48 ± 0.04	5.48 ± 0.04	21.8	21.8 ± 0.03	21.8 ± 0.01	1.74 ± 0.05	1.74 ± 0.08
#82	7.89 ± 0.11	7.92 ± 0.05	6.89	6.89 ± 0.01	6.89 ± 0.01	22.3	22.3 ± 0.01	22.3 ± 0.01	1.31 ± 0.03	1.31 ± 0.12
#83	7.36 ± 0.09	7.39 ± 0.06	NT	NT	NT	22.5	22.5 ± 0.01	22.5 ± 0.01	1.63 ± 0.07	1.63 ± 0.04
#84	35.3 ± 0.04	35.5 ± 0.05	NT	NT	NT	22.5	22.5 ± 0.01	22.5 ± 0.07	1.90 ± 0.04	1.90 ± 0.04
#85	7.15 ± 0.08	7.18 ± 0.04	10.04	10.0 ± 0.01	10.0 ± 0.02	22.6	22.5 ± 0.02	22.6 ± 0.02	1.31 ± 0.05	1.32 ± 0.03

#86	8.97 ± 0.11	9.01 ± 0.05	3.57	3.57 ± 0.01	3.56 ± 0.04	22.9	22.9 ± 0.02	22.9 ± 0.01	1.51 ± 0.05	1.52 ± 0.05
#87	7.19 ± 0.04	7.22 ± 0.05	NT	NT	NT	23.0	22.9 ± 0.03	23.0 ± 0.03	1.35 ± 0.03	1.35 ± 0.04
#88	6.24 ± 0.10	6.27 ± 0.05	4.83	4.83 ± 0.07	4.83 ± 0.07	23.4	23.4 ± 0.01	23.4 ± 0.03	1.13 ± 0.07	1.13 ± 0.03
#89	8.19 ± 0.15	8.23 ± 0.04	4.59	4.59 ± 0.02	4.59 ± 0.03	23.5	23.5 ± 0.01	23.5± 0.01	1.98 ± 0.06	1.98 ± 0.06
#90	7.12 ± 0.02	7.16 ± 0.05	7.75	7.75 ± 0.03	7.75 ± 0.04	24.6	23.3 ± 0.24	24.6 ± 0.20	1.06 ± 0.03	1.06 ± 0.03
#91	7.26 ± 0.13	7.30 ± 0.05	3.90	3.90 ± 0.01	3.87 ± 0.01	24.9	23.6 ± 0.24	24.8 ± 0.19	2.06 ± 0.03	2.05 ± 0.05
#92	5.98 ± 0.08	6.01 ± 0.07	7.67	7.67 ± 0.03	7.68 ± 0.01	26.2	26.2 ± 0.01	26.3 ± 0.02	1.98 ± 0.07	1.98 ± 0.11
#93	8.25 ± 0.10	8.29 ± 0.06	8.55	8.55 ± 0.01	8.54 ± 0.04	27.0	27.0 ± 0.01	27.1 ± 0.01	2.10 ± 0.03	2.10 ± 0.13
#94	7.43 ± 0.02	7.46 ± 0.06	NT	NT	NT	27.7	27.7 ± 0.01	27.7 ± 0.01	1.91 ± 0.06	1.91 ± 0.04
#95	11.5 ± 0.08	11.5 ± 0.04	NT	NT	NT	27.7	27.7 ± 0.06	27.7 ± 0.01	2.49 ± 0.08	2.49 ± 0.02
#96	9.14 ± 0.03	9.18 ± 0.05	NT	NT	NT	28.1	28.1 ± 0.02	28.1 ± 0.02	1.59 ± 0.04	1.58 ± 0.05
#97	9.85 ± 0.08	9.90 ± 0.10	7.27	7.27 ± 0.03	7.27 ± 0.04	28.3	28.3 ± 0.01	28.4 ± 0.01	1.84 ± 0.03	1.84 ± 0.05
#98	6.21 ± 0.09	6.24 ± 0.04	2.59	2.59 ± 0.12	2.60 ± 0.02	32.2	32.2 ± 0.03	32.1 ± 0.07	0.85 ± 0.03	0.84 ± 0.03
#99	7.99 ± 0.04	8.03 ± 0.06	13.7	13.7 ± 0.03	13.7 ± 0.02	39.3	39.3 ± 0.04	39.3 ± 0.03	1.59 ± 0.02	1.60 ± 0.08
#100	15.4 ± 0.08	15.4 ± 0.04	25.0	25.0 ± 0.02	25.1 ± 0.05	54.9	54.8 ± 0.02	54.9 ± 0.02	4.03 ± 0.02	4.03 ± 0.04
#101	21.5 ± 0.09	21.6 ± 0.04	2.70	2.70 ± 0.02	2.71 ± 0.01	NT	NT	NT	1.31 ± 0.06	1.31 ± 0.03
#102	9.86 ± 0.08	9.91 ± 0.02	3.12	3.12 ± 0.01	3.13 ± 0.10	NT	NT	NT	1.49 ± 0.03	1.49 ± 0.04
#103	6.58 ± 0.03	6.61 ± 0.04	3.17	3.16 ± 0.04	3.17 ± 0.03	NT	NT	NT	0.95 ± 0.06	0.95 ± 0.06
#104	6.78 ± 0.11	6.81 ± 0.05	4.06	4.06 ± 0.03	4.03 ± 0.01	NT	NT	NT	1.16 ± 0.08	1.16 ± 0.03
#105	18.9 ± 0.06	19.0 ± 0.06	4.11	4.11 ± 0.01	4.13 ± 0.01	NT	NT	NT	1.37 ± 0.04	1.36 ± 0.03
#106	6.49 ± 0.02	6.52 ± 0.05	4.31	4.31 ± 0.01	4.31 ± 0.04	NT	NT	NT	1.08 ± 0.05	1.08 ± 0.03
#107	7.01 ± 0.02	7.04 ± 0.13	4.40	4.40 ± 0.01	4.39 ± 0.05	NT	NT	NT	0.87 ± 0.04	0.88 ± 0.02
#108	5.95 ± 0.12	5.98 ± 0.10	4.70	4.70 ± 0.03	4.70 ± 0.01	NT	NT	NT	0.98 ± 0.07	0.98 ± 0.05
#109	25.2 ± 0.03	25.3 ± 0.01	4.80	4.80 ± 0.02	4.81 ± 0.02	NT	NT	NT	2.17 ± 0.04	2.17 ± 0.03
#110	8.17 ± 0.07	8.21 ± 0.06	4.90	4.90 ± 0.01	4.90 ± 0.08	NT	NT	NT	1.52 ± 0.10	1.52 ± 0.04
#111	20.1 ± 0.06	20.2 ± 0.07	4.90	4.90 ± 0.01	4.91 ± 0.01	NT	NT	NT	1.74 ± 0.07	1.74 ± 0.04
#112	15.5 ± 0.03	15.5 ± 0.03	4.95	4.95 ± 0.03	4.96 ± 0.03	NT	NT	NT	1.27 ± 0.04	1.27 ± 0.06
#113	15.3 ± 0.02	15.3 ± 0.13	5.20	5.20 ± 0.02	5.22 ± 0.01	NT	NT	NT	2.16 ± 0.04	2.16 ± 0.03
#114	9.34 ± 0.03	9.39 ± 0.02	7.11	7.11 ± 0.01	7.11 ± 0.01	NT	NT	NT	2.06 ± 0.02	2.08 ± 0.07
#115	10.2 ± 0.07	10.3 ± 0.07	7.45	7.45 ± 0.02	7.47 ± 0.02	NT	NT	NT	<LOQ	0.27 ± 0.02

#116	5.09 ± 0.04	5.11 ± 0.03	14.1	14.1 ± 0.01	14.1 ± 0.04	NT	NT	NT	<LOQ	0.42 ± 0.02
#117	20.4 ± 0.05	20.5 ± 0.13	20.1	20.1 ± 0.03	20.1 ± 0.02	NT	NT	NT	<LOQ	0.37 ± 0.04
#118	18.1 ± 0.04	18.2 ± 0.02	35.8	35.7 ± 0.04	35.8 ± 0.06	NT	NT	NT	1.23 ± 0.03	1.23 ± 0.08
**Sample ID**	**LR LC-MS (n = 3)**	**HR LC-MS (n = 3)**	**ELISA**	**LR LC-MS (n = 3)**	**HR LC-MS (n = 3)**	**ELISA**	**LR LC-MS (n = 3)**	**HR LC-MS (n = 3)**	**LR LC-MS (n = 3)**	**HR LC-MS (n = 3)**
Δ119	4.50 ± 0.03	4.53 ± 0.03	1.42	1.42 ± 0.10	1.42 ± 0.06	16.4	16.5 ± 0.14	16.5 ± 0.04	1.02 ± 0.07	1.02 ± 0.12
Δ120	5.16 ± 0.08	5.19 ± 0.03	1.64	1.64 ± 0.17	1.64 ± 0.07	18.6	18.2 ± 0.16	18.5 ± 0.19	1.07 ± 0.08	1.08 ± 0.04
Δ121	4.43 ± 0.03	4.46 ± 0.06	1.40	1.40 ± 0.07	1.40 ± 0.05	16.1	16.1 ± 0.31	16.1 ± 0.12	1.06 ± 0.08	1.06 ± 0.06
Δ122	3.73 ± 0.07	3.76 ± 0.02	1.23	1.23 ± 0.18	1.23 ± 0.21	13.6	13.3 ± 0.42	13.3 ± 0.37	0.86 ± 0.06	0.87 ± 0.10
Δ123	4.20 ± 0.01	4.22 ± 0.04	1.36	1.35 ± 0.12	1.35 ± 0.01	15.3	15.2 ± 0.41	15.3 ± 0.30	0.98 ± 0.07	0.98 ± 0.11
Δ124	3.58 ± 0.06	3.61 ± 0.02	1.80	1.80 ± 0.12	1.80 ± 0.05	13.1	13.1 ± 0.26	13.0 ± 0.30	<LOQ	0.56 ± 0.13
Δ125	3.86 ± 0.08	3.88 ± 0.03	1.39	1.39 ± 0.04	1.39 ± 0.01	14.0	13.9 ± 0.39	13.9 ± 0.38	0.92 ± 0.14	0.93 ± 0.09
Δ126	3.13 ± 0.03	3.15 ± 0.05	1.11	1.11 ± 0.10	1.11 ± 0.06	11.4	11.2 ± 0.28	11.2 ± 0.05	0.75 ± 0.05	0.75 ± 0.07
Δ127	4.08 ± 0.03	4.10 ± 0.02	1.33	1.33 ± 0.07	1.33 ± 0.03	14.9	14.8 ± 0.16	14.8 ± 0.18	0.82 ± 0.06	0.83 ± 0.10
Δ128	2.74 ± 0.02	2.75 ± 0.04	0.98	0.98 ± 0.05	0.98 ± 0.02	9.98	9.90 ± 0.20	9.90 ± 0.04	0.66 ± 0.15	0.67 ± 0.08
Δ129	3.60 ± 0.06	3.62 ± 0.07	1.22	1.22 ± 0.02	1.22 ± 0.02	13.1	12.8 ± 0.19	12.8 ± 0.01	0.66 ± 0.05	0.66 ± 0.07
Δ130	2.82 ± 0.01	2.84 ± 0.02	0.95	0.95 ± 0.05	0.96 ± 0.02	10.3	10.1 ± 0.31	10.1 ± 0.13	0.71 ± 0.08	0.72 ± 0.06
Δ131	2.44 ± 0.08	2.46 ± 0.02	0.90	0.90 ± 0.06	0.90 ± 0.05	8.49	8.30 ± 0.13	8.30 ± 0.01	0.67 ± 0.06	0.67 ± 0.09
Δ132	2.26 ± 0.07	2.28 ± 0.04	0.73	0.72 ± 0.03	0.72 ± 0.01	8.23	8.14 ± 0.43	8.14 ± 0.04	<LOQ	0.54 ± 0.07
Δ133	3.18 ± 0.03	3.20 ± 0.01	1.09	1.09 ± 0.01	1.09 ± 0.01	11.6	11.3 ± 0.03	11.3 ± 0.02	0.78 ± 0.06	0.79 ± 0.11
Δ134	2.59 ± 0.09	2.61 ± 0.03	0.91	0.91 ± 0.09	0.91 ± 0.06	9.43	9.26 ± 0.08	9.26 ± 0.09	0.64 ± 0.15	0.65 ± 0.06
Δ135	4.18 ± 0.01	4.21 ± 0.05	1.45	1.45 ± 0.05	1.45 ± 0.05	9.98	9.90 ± 0.23	9.90 ± 0.09	1.05 ± 0.08	1.05 ± 0.05
Δ136	3.79 ± 0.08	3.82 ± 0.03	1.34	1.34 ± 0.02	1.34 ± 0.02	13.8	13.6 ± 0.16	13.6 ± 0.03	0.96 ± 0.07	0.97 ± 0.04
Δ137	1.84 ± 0.13	1.85 ± 0.03	0.64	0.64 ± 0.03	0.64 ± 0.02	6.70	6.56 ± 0.12	6.56 ± 0.01	<LOQ	0.59 ± 0.08
Δ138	1.51 ± 0.11	1.52 ± 0.04	0.48	0.48 ± 0.04	0.48 ± 0.02	5.50	5.30 ± 0.09	5.30 ± 0.05	<LOQ	0.42 ± 0.09
Δ139	3.09 ± 0.03	3.11 ± 0.06	1.05	1.04 ± 0.16	1.04 ± 0.16	11.2	11.0 ± 0.34	11.2 ± 0.33	0.78 ± 0.06	0.78 ± 0.08
Δ140	3.47 ± 0.05	3.49 ± 0.03	1.11	1.11 ± 0.02	1.11 ± 0.03	12.6	12.5 ± 0.11	12.5 ± 0.10	0.78 ± 0.09	0.79 ± 0.06
Δ141	2.89 ± 0.21	2.91 ± 0.04	1.06	1.06 ± 0.02	1.06 ± 0.02	10.5	10.3 ± 0.15	10.3 ± 0.09	0.73 ± 0.04	0.74 ± 0.05
Δ142	2.24 ± 0.06	2.25 ± 0.04	0.81	0.81 ± 0.03	0.81 ± 0.01	8.14	8.05 ± 0.36	8.05 ± 0.27	<LOQ	0.56 ± 0.07
Δ143	1.76 ± 0.03	1.77 ± 0.03	0.61	0.61 ± 0.02	0.61 ± 0.01	6.42	6.31 ± 0.15	6.31 ± 0.17	<LOQ	0.48 ± 0.08

Δ144	2.66 ± 0.02	2.68 ± 0.08	1.12	1.12 ±0.09	1.12 ± 0.10	9.70	9.48 ± 0.24	9.48 ± 0.07	0.77 ± 0.06	0.77 ± 0.07
Δ145	2.90 ± 0.02	2.92 ± 0.04	1.09	1.09 ± 0.02	1.09 ± 0.01	10.6	10.5 ± 0.07	10.5 ± 0.02	0.71 ± 0.02	0.72 ± 0.04
Δ146	2.44 ± 0.18	2.45 ± 0.02	0.90	0.90 ± 0.04	0.90 ± 0.02	8.87	8.73 ± 0.25	8.73 ± 0.02	0.67 ± 0.05	0.67 ± 0.05
Δ147	2.45 ± 0.08	2.47 ± 0.02	1.06	1.06 ± 0.04	1.06 ± 0.03	8.92	8.73 ± 0.35	8.73 ± 0.09	0.73 ± 0.05	0.73 ± 0.08
Δ148	1.91 ± 0.04	1.92 ± 0.03	0.61	0.61 ± 0.01	0.61 ± 0.01	6.96	6.88 ± 0.24	6.88 ± 0.09	<LOQ	0.53 ± 0.05
Δ149	2.92 ± 0.02	2.94 ± 0.01	1.02	1.02 ± 0.05	1.02 ± 0.02	10.6	10.5 ± 0.15	10.5 ± 0.17	0.76 ± 0.10	0.76 ± 0.07
Δ150	1.85 ± 0.14	1.86 ± 0.01	0.74	0.73 ± 0.04	0.73 ± 0.04	6.73	6.51 ± 0.10	6.51 ± 0.02	<LOQ	0.56 ± 0.07
Δ151	2.57 ± 0.02	2.58 ± 0.02	0.99	0.99 ± 0.01	0.99 ± 0.01	9.34	9.26 ± 0.38	9.26 ± 0.01	0.75 ± 0.06	0.75 ± 0.09
Δ152	2.53 ± 0.09	2.54 ± 0.04	0.95	0.95 ± 0.01	0.95 ± 0.01	9.20	9.12 ± 0.13	9.12 ± 0.03	0.74 ± 0.03	0.75 ± 0.12
Δ153	3.29 ± 0.09	3.31 ± 0.06	1.00	1.00 ± 0.01	1.00 ± 0.01	12.0	11.9 ± 0.15	11.9 ± 0.09	0.73 ± 0.04	0.74 ± 0.08
Δ154	3.01 ± 0.04	3.03 ± 0.02	0.89	0.89 ± 0.03	0.89 ± 0.03	11.0	10.7 ± 0.05	10.7 ± 0.05	0.68 ± 0.06	0.68 ± 0.04
Δ155	1.38 ± 0.12	1.38 ± 0.05	0.61	0.61 ± 0.01	0.61 ± 0.01	5.00	4.88 ± 0.11	4.89 ± 0.12	<LOQ	0.41 ± 0.13
Δ156	1.82 ± 0.10	1.83 ± 0.03	0.61	0.61 ± 0.01	0.61 ± 0.01	6.63	6.56 ± 0.10	6.56 ± 0.12	<LOQ	0.48 ± 0.05
Δ157	2.23 ± 0.13	2.25 ± 0.07	0.92	0.92 ± 0.01	0.92 ± 0.01	8.14	8.03 ±0.09	8.03 ± 0.04	<LOQ	0.58 ± 0.06
Δ158	2.67 ± 0.06	2.68 ± 0.03	1.01	1.01 ± 0.04	1.01 ± 0.02	9.71	9.62 ± 0.12	9.62 ± 0.07	0.66 ± 0.12	0.66 ± 0.09

NT = not tested.
